# The Recurrent Vulvovaginal Candidiasis: Proposal of a Personalized Therapeutic Protocol

**DOI:** 10.5402/2011/806065

**Published:** 2011-08-09

**Authors:** F. Murina, A. Graziottin, R. Felice, G. L. Radici, S. Di Francesco

**Affiliations:** ^1^Outpatient Department of Vulvar Disease, V. Buzzi Hospital, Via Castelvetro 32, 20124 Milan, Italy; ^2^Center of Gynecology, San Raffaele Resnati Hospital, 20124 Milan, Italy

## Abstract

*Background*. Recurrent vulvovaginal candidiasis (RVC) is an increasing challenge in clinical practice. *Objective*. The purpose of this study was to reduce the episodes of RVC through the intake of fluconazole 200 mg/dose with a personalized regimen at growing administration intervals with a probiotic. *Method*. 55 patients received a 200 mg fluconazole as an induction dose for 3 alternate days. Symptoms resolution after 10–14 days made the patients eligible to continue with a maintenance therapy of fluconazole weekly for one month, followed by 200 mg after 10, 15, 20 and 30 days. Patients were allowed to move on to the next level of maintenance therapy only if they were symptom free. Patients were also given a probiotic with Beta Glucan and Echinacea Purpurea. *Results*. Among the 55 patients enrolled, four (7%) have withdrawn after the induction phase. 51 patients completed the whole therapeutic maintenance period, and eight (15,6%) experienced a recurrence before the end of the therapy. Five women (9,8%) relapsed (two after 2 months and three after 6 months). *Conclusion*. The positive results of our study prove the effectiveness of an individualized protocol for a rather short period, with a slowly decreasing administration of fluconazole + probiotic.

## 1. Introduction

The mycotic vulvovaginitis is a common infection. It is estimated that about 75% of women will experience this infection at least once during their life time. In 15% of these cases the mycotic infection may evolve in a “cyclic recurrent type” (RVC) defined as four or more episodes of mycotic vulvovaginitis during one year [1, 2]. Over 85% of cases are caused by Candida Albicans, while Candida Tropicalis and Glabrata are more common in diabetic women.

The VVC has a negative effect on women's personal confidence and self-esteem [3]. It may cause or contribute to psycosexual problems, namely, introital dyspareunia, with progressive loss of genital arousal (pain is the strongest reflex inhibitor of vaginal congestion and lubrication), secondary loss of desire, and avoidance of sexual intimacy for fear of experiencing pain and recurrence of another Candida vulvovaginitis. It may as well cause discomfort, uneasiness, sense of shame, or unworthiness in informing both doctor and partner about the inconveniences of such a recurring infection.

An antimycotic prophylaxis with fluconazole for long periods of time has proven to be effective for the prevention of mycotic recurrent episodes.

The oral weekly administration of a single dose of 150 mg of fluconazole for a period of 6 months proved to be successful in 91% of the cases but decreased to 43% during the observation period 6 months after the cessation of the therapy. This stresses the need to maintain the fluconazole therapy for long periods of time [4]. 

Another regimen with a personalized and decreasing administration of 200 mg fluconazole doses, always for rather long periods (4 months on average), has been proposed. This new therapeutic scheme has shown no evidence of clinical recurrence in 90% of women, and reduced to 70% after 1 year [5].

The intestinal milieu is Candida's main “reservoir” and leads to a fungal colonization in the perineum-perianal and vaginal areas [6].

Probiotics are autochthonous microorganisms that populate the human gut since our birth and contribute to the maintenance of the intestinal ecosystem [7].

The objective of our study has been to reduce the episodes of RVC through the intake of fluconazole 200 mg/dose with a personalized regimen at growing administration intervals together with a special probiotic preparation with Beta Glucan and Echinacea Purpurea that develop immunomodulatory and antimycotic activity.

## 2. Materials and Methods

### 2.1. Patients Selection

Patients were eligible to be included in the study only if (1) they had experienced at least 4 documented episodes of RVC in the previous 12 months; (2) had a symptomatic vulvovaginal candidiasis attack at the moment of the enrollment; had the immunologic positivity of anticandidiasis antibodies found in the vaginal discharges through the Savvycheck tester.

The severity of symptoms has been evaluated through the symptomatologic score of Sobel [5]. In short, symptoms like pruritus, erythema, burnings, and oedema have been scored on a semiquantitative scale: 0 (absent), 1 (light), 2 (moderate), 3 (severe). A total score of ≥4 would have been considered as a case of symptomatic vulvovaginal candidiasis.

We did not include in the study women in pregnancy, diabetics, and those who had been treated with an antibiotic therapy in the previous two weeks. Patients were asked not to use any antibiotics or antimycotics during the study period.

### 2.2. Therapeutic Protocol

All patients received a 200 mg fluconazole orally as an induction dose for 3 alternate days during the first treatment week. Patients have been checked and evaluated after 10–14 days, and the evidence of symptoms resolution (Sobel score <4) and the negativity of Savvycheck test made them eligible to continue with a maintenance therapy of 200 mg fluconazole following the protocol therapy shown in Table 1.

In the case of recurrent vulvovaginal candidiasis (Sobel score ≥4) the protocol has been personalized suspending the growing intervals of the fluconazole dose, repeating the induction phase (fluconazole 200 mg dose 3 times in 1 week) and restarting with the original protocol later on.

Together with the systemic antimycotic therapy, a probiotic preparation, with Beta Glucan and Echinacea Purpurea, has been associated. The probiotic preparation has been produced with a “fast-slow dissolution” international patent that grants an efficient gastroprotection and the safe arrival of a high quantity of a specific selection of milkenzymes in the intestinal “reservoir”. Patients were given 3 tablets a day during the first week and 2 tablets a day for the following 2 months.

Patients were evaluated after 2 and 6 months from the end of the maintenance protocol. 

The clinical outcome was classified and recorded as follows:

- “optimal responders”: all women that completed the therapy scheme and never experienced a new recurrence during the follow-up period;
- “suboptimal responders”: the patients that experienced up to 2 episodes of syntomatologic vulvovaginal recurrence during maintenance or follow-up period;
- “poor responders”: patients that experienced 3 or more episodes of vulvovaginal candidiasis during maintenance or follow-up periods.

The statistical analysis has been worked out with a Microsoft Excel 2007 programme.

## 3. Results

All the 55 patients enrolled (average age 29, range 21–44) have been included in the study; among them 4 (7%) have withdrawn from the trial after the induction phase with fluconazole + probiotic for the symptomatologic persistence and the positivity of the Savvycheck test.

Among the 51 patients that completed the whole therapeutic maintenance eight (15,6%) experienced a vulvovaginal recurrence before the end of the therapy and restarted the protocol from the induction phase.

Once completed the fluconazole + probiotic therapy, during the follow-up period only five women (9,8%) experienced symptomatologies that could be related to RVC, in particular two after 2 months and three after 6 months.

## 4. Discussion

The positive results of our study prove the effectiveness of a personalized protocol for a rather short period, with a slowly decreasing administration of fluconazole + probiotic as a first line therapy for the RVC.

Candida can be part of the vaginal ecosystem as a minority germ, mostly in the dormant form of spora and/or can reach the vaginal lumen mainly through the perianal area [8]. Once arrived, it starts the colonization creating an avirulent phase as a vaginal host.

Gut can be considered one of the main sources of candidiasis colonization in the vaginal area, even if his exact role in the recurrent vulvovaginitis is still under discussion.

Candida isolated from rectal cultures of patients with RVC has been shown to be exactly the same of the one found in vaginal areas, supporting the hypothesis of the existence of a consistent intestinal reservoir of the fungus [6]. 

It is possible to identify two different infections of the candidiasis in the vulvovaginal area: one being the “classic” infection, a severe type white dense leakage with only occasional recurrences and the other a cyclical type with several recurrences almost on a monthly basis.

The emerging hypothesis is that these two forms stem from a different host reaction, modulated by the germ: in the severe type, the candidiasis develops an immunosuppressive action being present in high quantities, while in the cyclic-recurrent type it generates an hypersensitivity reaction acting with a “small quantity” in subjects with genetic predisposition [9–11]. 

An increasing body of evidence can support the definition of RVC being an allergic irritating dermatitis as a consequence of the reaction to small quantities of Candida Albicans in subjects with genetic predisposition.

The individual reaction is linked to endogenous substances that modulate the tissue response, resulting from the interaction between mast cells and substances like interleukins and the mannose-binding lectin (MBL) involved in mycotic infections. Furthermore, a constitutive deficiency of the inflammasomes type NALP3, macromolecules that govern the production and the release of interleukins (IL) 1*β*, has been evidenced [12]. 

Consequently, in VVC we can introduce the concept of “*vaginal cutoff*” of candidiasis that is the minimum quantity-level of the fungus above which it would activate a reaction of allergic type that justifies the cyclic-recurrent episodes of vulvovaginal candidiasis, in vulnerable subjects.

Sobel et al. in the ReCiDif trial [4, 5] have proven that the therapy of a RVC needs a long period of antimycotic prophylaxis (6–12 months), but they also showed that the rate of recurrences after the suspension of the fluconazole administration remains quite high regardless of the total medicine dose used (Sobel et al.: 3.9 g versus ReCiDif trial: 4 g). A personalized treatment with a fluconazole decreasing-doses regimen (ReCiDif trial) has shown a lower rate of recurrences compared to the weekly rigid protocol implemented in a nonstop system for 6 months [4, 5].

In our opinion the fundamental element is the capability to keep the vaginal candidiasis just below the cut-off threshold level, above which it would unleash the inflammatory events on allergic base typical of the RVC.

Fluconazole is absolutely suitable for this purpose both for the tolerability and the pharmaceutical profile; it has a 90% bioavailability with an action profile that keeps the therapeutic efficiency up to 5 days [13].

The therapeutic dose determines a quantity of active ingredient sufficient to prevent the development of a candidiasis infection that, as we said, is linked to a high growth of the fungus with an immunosuppressive action.

In the RVC, the fungus amount is split in small quantities therefore it is realistic to consider that the fluconazole efficacy can last for longer periods. This possibility is increased even further by the use of the 200 mg medicine dose.

We believe that the personalized posological scheme with decreasing doses, that was shown to be more efficient than the weekly rigid one, should be the leading element in the protocol therapy of the RVC.

The vaginal ecosystem has to gradually adapt itself to the capability of keeping the balance between the candidiasis level just below the cut-off threshold, to avoid the unleash of the vulvovaginal allergic reaction, and the immunoregulating vaginal elements that are responsible of the inflammatory cascade (lymphokines, cytokines, MBL, vaginal epithelial cells, mast cells etc.).

The positive results of our study suggest that the 200 mg fluconazole tablet does not necessarily have to be taken for long periods but can be assumed at progressive longer intervals of time in order to develop a kind of “gradual hyposensitization”.

In fact, the concept of “hyposensitization” takes into account the reduction of the sensibility towards the allergen in order to keep it as low as possible and make it harmless.

Being able to keep the vaginal candidiasis quantity to a nonreaction level would allow women to develop a mechanism with such an immunoregulating effect to prevent the fungus growth above the critical threshold once the fluconazole administration is suspended.

To integrate the fluconazole treatment with a valid probiotic is another critical step, as we believe it may promote a synergist adjuvant effect to restore the homeostasis of the vagina-intestine area that is usually severely disregulated in women with RVC.

Probiotics like Saccharomyces c. and Bifidobacterium L. are special autochthon elements that populate the human gut since the day we were born. They contribute to maintenance of the intestinal ecosystem and modulate the inflammatory reaction of the T helper lymphocytes [14].

A randomised clinical trial with placebo versus probiotics such as Lactobacillus acidophilus, L. rhamnosus GR-1, and L. fermentum RC-14 has shown their efficacy in reducing the vaginal candidiasis colonization [15].

The peculiar probiotic we decided to use in the study has two characteristics: a specific milk enzymes selection of different families and an important quantity of Beta Glucan from Saccharomyces cerevisiae that has an effective immunoregulating activity.

This special probiotic is manufactured with a patent “fast-slow dissolution” technology that grants a valid gastroprotection to the active microorganism in order to let them reach the gut to be able to colonize and balance the ecosystem.

The promising results of the study have certain limitations due to a nonelevated number of patients and a rather short follow-up period. Moreover, the therapeutic weight of the probiotic used in addition to the antimycotic therapy needs to be more carefully evaluated.

Nevertheless, the positive results encourage implementing rigorous therapeutic protocols that can be personalized in order to achieve the highest efficacy against the aetiopathogenetic mechanism of the RVC.

## Figures and Tables

**Table 1:** Therapeutic protocol with fluconazole 200 mg + special probiotic.

Fluconazole 200 mg: 1 tbl as an induction dose for 3 alternate days during the first treatment week (total 3 tbls)	Probiotic: 1 tbl 3 times a day for 1 week
Fluconazole 200 mg: 1 tbl a week for 4 weeks	

Fluconazole 200 mg: 1 tbl after 10 days	
Fluconazole 200 mg: 1 tbl after 15 days	Probiotic: 1 tbl 2 times a day for 8 weeks
Fluconazole 200 mg: 1 tbl after 20 days	
Fluconazole 200 mg: 1 tbl after 30 days then STOP	
